# The Effects of Sociodemographic Factors and BMI on Weight Stigma Among Adults With Obesity in Madinah Region, Saudi Arabia: A Cross-Sectional Analytical Study

**DOI:** 10.7759/cureus.63993

**Published:** 2024-07-06

**Authors:** Saeed S Alammari, Mohammed A Almatrafi, Jebreel M Fallatah, Khalid F Alahmadi, Yousef A Aloufi, Abdullah S Alahmadi

**Affiliations:** 1 Preventive Medicine, Al Madinah Health Cluster, Ministry of Health, Madinah, SAU; 2 Family Medicine, Al Madinah Health Cluster, Ministry of Health, Madinah, SAU

**Keywords:** saudi arabia, body mass index (bmi), weight-related stigma, al-madinah region, public health, obesity, weight self-stigma

## Abstract

Introduction

Weight stigma (WS), characterized by discrimination and stereotyping based on a person’s weight, remains understudied in Saudi Arabia despite the country’s high obesity rates. Particularly, limited research has been conducted on WS in Madinah. Understanding the factors contributing to WS in this region is crucial for developing targeted interventions to effectively address it. Hence, this study aimed to explore the effects of sociodemographic characteristics and body mass index (BMI) on WS among adults with obesity in Madinah.

Methods

Individuals with obesity who were seeking care at primary healthcare centers were included in this study. This research was an analytical cross-sectional study; Madinah City was divided into four areas. One primary health center from each area was randomly selected. Subsequently, a consecutive sampling technique was used to collect questionnaires from participants during the period of December 2023 to March 2024. The participants completed a self-administered electronic questionnaire, which included the Arabic-translated and validated version of the Weight Self-Stigma Questionnaire (WSSQ). Data analysis included descriptive, simple logistic regression and multiple logistic regression with forward stepwise analysis.

Results

A total of 383 participants completed the questionnaire, of which 225 (58.7%) were men and 158 (41.3%) were women. The analysis showed that individuals without a family history of obesity experienced higher WS levels than those with a family history [adjusted odds ratio (AOR) = 1.853, 95% confidence interval (CI): 1.010-2.844]. Moreover, individuals with obesity demonstrated the lowest WS levels than those without obesity (AOR = 0.027, 95% CI: 0.009-0.08). These findings provide insights into the association among sociodemographic factors, BMI, and WS in adults with obesity residing in Madinah, Saudi Arabia.

Conclusion

This study provides evidence that WS is a complex issue that is not solely determined by an individual’s obesity status; rather, it is influenced by a lack of family history of obesity, which establishes the impact of social factors on WS. Therefore, comprehending the role of family dynamics and societal norms in shaping an individual's weight status is crucial in managing WS.

## Introduction

Weight stigma (WS), also known as weight bias or weight discrimination, is described as “discrimination or stereotyping based on a person’s weight” [1]. Weight self-stigma refers to feelings of shame, unfavorable self-evaluations, and perceived discrimination.

Enacted stigma corresponds to the direct experience of prejudice in several aspects of social existence, such as employment, social interaction, limited access to public services, and housing. Self-stigma occurs when an individual internalizes negative beliefs and feelings about themselves due to their association with a stigmatized group. This includes devaluing themselves and experiencing fear of being treated negatively by others based on this stigma [2].

The stigma associated with obesity is characterized by prejudiced, stereotypical, and discriminatory attitudes and behaviors against individuals who are obese. These attitudes and behaviors are often driven by misconceptions about the causes of obesity. In most societies, obesity is often viewed as a result of personal failings or weaknesses [3].

In addition, weight-related stigma can result in the internalization of experiences that exert a negative impact on one’s quality of life in general [4]. Our knowledge regarding the factors that contribute to weight self-stigma or the internalization of weight-related attitudes and beliefs by individuals with obesity is limited.

Obesity is considerably prevalent in Saudi Arabia, with 43.5% of men and 34.7% of women being overweight. The obesity rate is currently at 23.5% for women and 23.9% for men [5]. WS has been reported to be linked to an increased prevalence of depressive symptoms and decreased psychological well-being as it has a negative impact on individuals [6].

A previous study indicated that women tend to exhibit less weight-stigmatizing attitudes compared to men. Furthermore, individuals in late middle age are less likely to hold weight-stigmatizing views than their younger or older counterparts. Notably, these attitudes are positively correlated with income, with the most pronounced stigmatization occurring within the intermediate education and occupational social classes [7]. WS appears to be more prevalent among younger individuals and is linked to various factors such as marital status, occupation, higher body mass index (BMI), and personal experiences of weight-related stigma [8]. A study conducted by Hughes et al. highlights that WS is particularly pronounced among females and those from socioeconomically disadvantaged backgrounds. Additionally, this study found that while higher BMI during childhood, adolescence, and early adulthood is associated with increased WS, BMI alone does not fully account for these differences, suggesting the influence of additional factors [9].

Research on WS related to BMI is lacking in Saudi Arabia despite the high prevalence of obesity in the country. Publications on the prevalence of WS in Saudi Arabia are limited, and specific studies have not been conducted in the region of Madinah. Hence, performing such a study in this city would be valuable in identifying the specific factors contributing to WS in the area. This knowledge is crucial for developing targeted interventions and strategies to effectively address WS. By understanding the unique characteristics in the local context, researchers, policymakers, and healthcare professionals can design culturally tailored interventions that are necessary and are likely to achieve positive outcomes in alleviating this stigma in Madinah.

To develop effective interventions aimed at reducing WS, a thorough understanding of WS is essential. Measures that effectively reduce the stigma associated with weight should be implemented as these can significantly improve the overall health and well-being of individuals categorized as obese. Achieving this goal requires a precise understanding of the specific factors that make certain individuals susceptible to experiencing WS. This study aimed to explore the effects of sociodemographic characteristics and BMI on WS among adults with obesity in the Madinah region.

## Materials and methods

Study design and participants

This was an analytical cross-sectional study carried out in governmental primary healthcare centers (PHCs) in Madinah. Al-Madinah Al-Munawarah, located in the Western Province of Saudi Arabia, is the main region of the country with a total population of 2,239,923 [10]. Individuals who were visiting the PHC were the target population. We included all Individuals aged ≥18 years, both sexes, living in the Madinah region, who attended the PHC for any health issue.

Sampling method and sample size

Madinah City was divided into four geographic areas: south, north, west, and east. We used a random sampling method to select one Ministry of Health PHC from each area to ensure the sample was representative of these different regions. Following the selection of the PHCs, consecutive sampling was utilized to collect the distributed questionnaires of individuals attending these PHCs between December 2023 and March 2024. 

A study previously conducted by Althumiri et al. revealed that 43.7% of individuals living in Madinah experienced WS [11]. In this study, the minimum required sample size of 379 was determined using the sample size formula (n = Z2pq/E2), considering a 95% confidence interval (CI) and a margin of error of ±5%.

Data collection

A self-administered electronic questionnaire translated into Arabic and validated in a prior study conducted by BinDhim et al. was used to collect data after obtaining electronic informed consent from each participant [12]. To enable anonymous and voluntary participation, an electronic questionnaire was generated using Google Forms and was made accessible to be filled out during the study. To ensure the accuracy of the data, the questionnaire replies were completed in an Excel sheet that was electronically connected to Google Forms.

Anthropometric data, including weight and height, were obtained from the electronic records of PHCs. To ensure accuracy, the data collector verified the respondents’ self-reported measurements by comparing them with the information in the PHC record and BMI was calculated. Furthermore, the data collector confirmed that the measurements of all individuals were within a typical, expected range. Values outside this range might have been inaccurate. This strict quality control process was implemented to uphold the accuracy and reliability of the anthropometric data collected for analysis. Inconsistencies or outliers were carefully reviewed to preserve the integrity of the dataset.

Instruments and variables

Sociodemographic Variables

In the first section, the survey included questions about participants’ demographic characteristics, such as age, sex, marital status (single, married), employment status (student, unemployed, currently employed, retired), education level (middle school or below, high school, bachelor’s degree, or postgraduate degree), monthly income (in Saudi Riyals, 0−999, 1000−4999, 5,000−9,999, 10,000−15,000, and >15,000), and type of housing (homeowner, renter, or living with family). In addition, family history of obesity (yes or no), whether the participant had previously attempted to lose weight in the last year (yes or no), and smoking history (current smoker, non-smoker, or previous smoker) were enquired.

The BMI was used to identify individuals who were generally overweight or obese. People with a BMI of >25 kg/m^2^ were considered overweight, whereas those with a BMI of >30 kg/m^2^ were classified as obese. Obesity was further categorized into three grades: Grade I included individuals with a BMI of 30-35, Grade II included those with a BMI of 35-40, and Grade III comprised those with a BMI of ≥40. Occasionally, Grade III obesity was referred to as “severe” obesity [13].

Weight Self-Stigma Questionnaire

In the second part of the survey, the levels of weight self-stigma were evaluated using a questionnaire called the Weight Self-Stigma Questionnaire (WSSQ) [14]. The WSSQ comprises 12 items categorized into two sections: one assessing self-devaluation and the other examining enacted stigma. The participants used a five-point Likert scale, selecting from options that ranged from ‘strongly disagree’ (1) to ‘strongly agree’ (5) to indicate their agreement level. Elevated scores in the self-devaluation section signified stronger negative attitudes and emotions regarding obesity. Cronbach α coefficients were found to be acceptable for the entire scale (α = 0.878) by validation of the questionnaire by Lillis et al. [14]. This study used the validated Arabic version of the questionnaire, with a Cronbach's α coefficient of (α = 0.898) for the overall survey [12].

A previous study has established that the Arabic version of the WSSQ is a reliable tool for evaluating weight-related self-stigma among Arabic-speaking individuals (Saudi nationals) [11]. Owing to the lack of a set cutoff point for the WSSQ, categories were created based on the median score. The scores were categorized into ‘lower WS’ for scores below the median (0 = total median score) and ‘higher WS’ for those above the median (1 = total median score).

Statistical analysis

The data was manually verified and then coded in an Excel sheet. To ensure accuracy, we used double-entry verification. We identified and resolved any discrepancies through comparison after two individuals independently entered the same data. To assess the association between variables, Descriptive statistics were used to estimate obesity prevalence, the Chi-squared test (χ2) was utilized, and quantitative data were presented as numbers and percentages.

A univariate analysis was performed to determine the factors associated with weight self-stigma, sociodemographic characteristics, and BMI, considering a p-value of <0.05 as being statistically significant. Before developing the final regression model, the data were screened for multicollinearity. This involved using linear regression diagnostics and Pearson’s correlation coefficient tests, highly correlated predictors were removed. To identify the factors linked to elevated weight self-stigma, Multiple logistic regression was conducted using a forward stepwise method, with model selection based on the likelihood-ratio test and criteria for entry set at 0.05 and for removal at 0.10; all demographic variables and BMI were incorporated. IBM SPSS Statistics for Windows, Version 25 (Released 2017; IBM Corp., Armonk, New York, United States) was used for the statistical analysis.

Ethical consideration

The study had been ethically approved by Health Ethics Committee of General Directorate of Health Affairs in Madinah, Saudi Arabia (approval number 23-105, dated November 26, 2023). The electronic informed consent of each participant was obtained. Ethical measures were also implemented to ensure the confidentiality and privacy of the collected data.

## Results

Demographic characteristics

**Figure 1:**
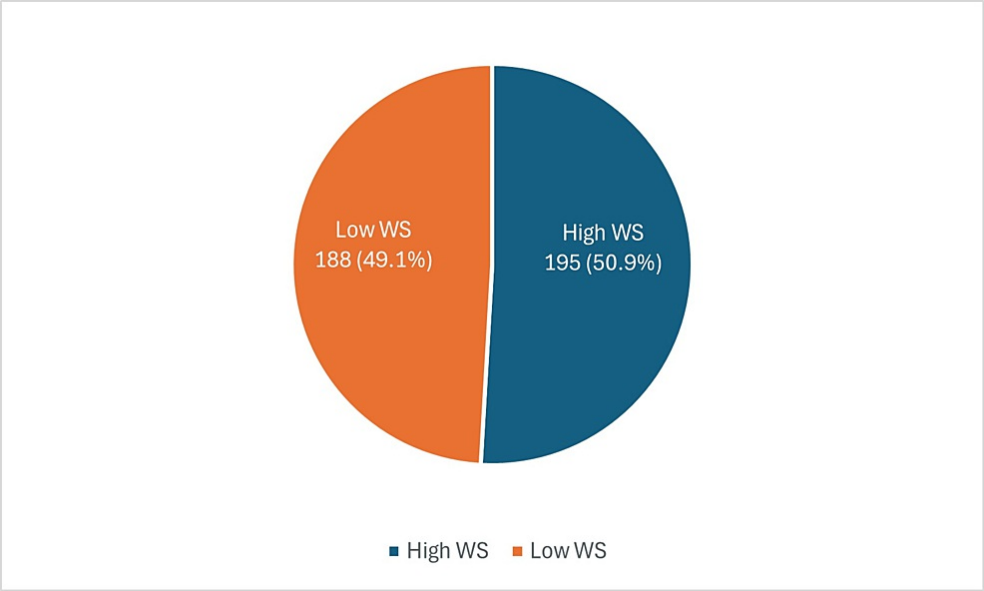
Overall prevalence of WS among participants WS: Weight stigma

A total of 383 participants completed the questionnaire, and Table 1 presents the key demographic characteristics of the respondents. Of the participants 225 (58.7%) were men and 158 (41.3%) were women. Moreover, 121 (31.6%) of the respondents were between 20 and 29 years of age, and 83 (21.7%) were between 30 and 39 years of age. Regarding educational qualifications, 200 (52.2%) of the participants had a bachelor’s degree and 137 (35.8%) had completed high school. When the employment status was enquired, 177 (46.2%) of the respondents stated that they were currently employed. Furthermore, 261 (68.1%) of the participants reported having a family history of obesity, and 219 (57.2%) had attempted weight loss in the past year. The prevalence of overweight was 73 (19.1%) and obesity class I was 74 (19.3%). Obesity class II was more prevalent 102 (26.6%) than other classes of obesity. The overall prevalence of WS in Madinah was 195 (50.9%) (Figure 1).

**Table 1:** Baseline characteristics of participants (n=383)

Characteristics		n (%)
Gender	Female	158 (41.3%)
	Male	225 (58.7%)
Age groups	18 - 19	68 (17.8%)
	20 -29	121 (31.6%)
	30 - 39	83 (21.7%)
	40 - 49	63 (16.4%)
	+ 50	48 (12.5%)
Obesity	Non-obese	51 (13.3%)
	Overweight	73 (19.1%)
	Obesity I	74 (19.3%)
	Obesity II	102 (26.6%)
	Obesity III	83 (21.7%)
Age onset of obesity	0 - 10	121 (31.6%)
	11 - 19	118 (30.8%)
	+ 20	144 (37.6%)
Employment status	Currently	177 (46.2%)
	Retired	19 (5.0%)
	Student	119 (31.1%)
	Unemployment	68 (17.8%)
Education	Middle school and below	26 (6.8%)
	High school	137 (35.8%)
	Bachelor's degree	200 (52.2%)
	Postgraduate	20 (5.2%)
Marital status	Single	189 (49.3%)
	Married	194 (50.7%)
Previous attempts to lose weight	No	164 (42.8%)
	Yes	219 (57.2%)
Family history of obesity	No	122 (31.9%)
	Yes	261 (68.1%)
Housing	Homeowner	66 (17.2%)
	Living with family	191 (49.9%)
	Renter	126 (32.9%)
Income (monthly)	0−999	58 (15.1%)
	1000−4999	54 (14.1%)
	5000−9999	95 (24.8%)
	10000−15000	107 (27.9%)
	over 15000	69 (18.0%)
Smoking status	No	279 (72.8%)
	Previously	30 (7.8%)
	Yes	74 (19.3%)
Total		383 (100%)

Weight stigma

**Figure 2:**
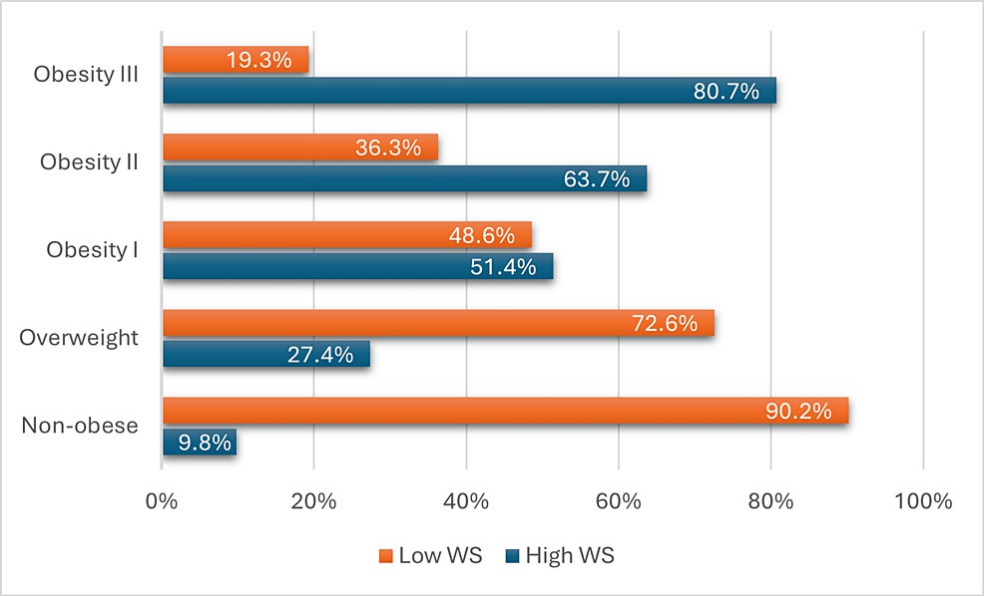
Distribution of high WS and low WS across different BMI categories WS: Weight stigma

**Figure 3:**
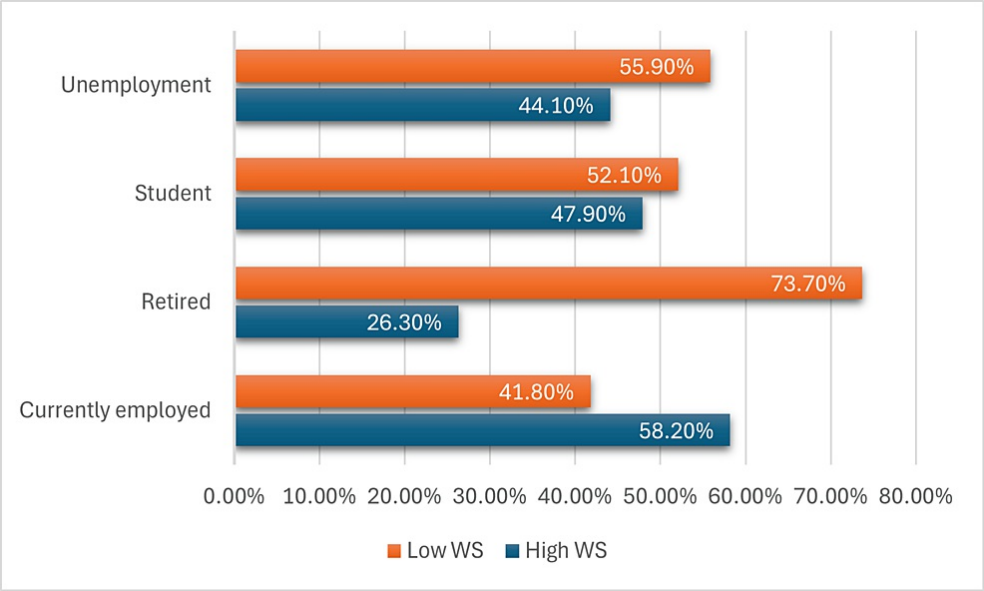
Distribution of high WS and low WS according to employment status WS: Weight stigma

The association of WS with various sociodemographic factors and BMI was examined. The findings indicated the absence of statistically significant differences in WS between men and women (p-value = 0.258). However, individuals aged 20-29 years showed a 121 (31.6%) higher prevalence of WS (p-value = 0.022) as shown in (Table 2); moreover, in the Obesity III category, 67 (80.7%) have high WS and 16 (19.3%) have low WS (Figure 2). Regarding employment status, currently employed individuals exhibit the highest proportion of high WS at 103 (58.2%), while retired individuals have the lowest proportion at 5 (26.3%) (Figure 3). Another key finding was that WS was significantly linked to employment status; people who were unemployed had a lower odd of WS (odds ratio (OR):0.012, 95% confidence interval (CI) =1.345-11.293) compared to employed individuals (Table 3).

**Table 2:** Prevalence of WS according to participant demographic characteristics *Significant values < 0.05 WS: Weight stigma

Variable		High WS	Low WS	Total	Chi square p-value
		n (%)	n (%)	n (%)	
Gender	Female	75 (47.5%)	83 (52.5%)	158 (41.3%)	0.258
	Male	120 (53.3%)	105 (46.7%)	225 (58.7%)	
Age groups	18 - 19	26 (38.2%)	42 (61.8%)	68 (17.8%)	0.022^*^
	20 -29	67 (55.4%)	54 (44.6%)	121 (31.6%)	
	30 - 39	47 (56.6%)	36 (43.4%)	83 (21.7%)	
	40 - 49	37 (58.7%)	26 (41.3%)	63 (16.4%)	
	+ 50	18 (37.5%)	30 (62.5%)	48 (12.5%)	
Obesity	Non obese	5 (9.8%)	46 (90.2%)	51 (13.3%)	<0.001*
	Overweight	20 (27.4%)	53 (72.6%)	73 (19.1%)	
	Obesity I	38 (51.4%)	36 (48.6%)	74 (19.3%)	
	Obesity II	65 (63.7%)	37 (36.3%)	102 (26.6%)	
	Obesity III	67 (80.7%)	16 (19.3%)	83 (21.7%)	
Age onset of obesity	0 - 10	74 (61.2%)	47 (38.8%)	121 (31.6%)	0.017^*^
	11 - 19	58 (49.2%)	60 (50.8%)	118 (30.8%)	
	+ 20	63 (43.8%)	81 (56.3%)	144 (37.6%)	
Employment status	Currently	103 (58.2%)	74 (41.8%)	177 (46.2%)	0.018^*^
	Retired	5 (26.3%)	14 (73.7%)	19 (5.0%)	
	Student	57 (47.9%)	62 (52.1%)	119 (31.1%)	
	Unemployment	30 (44.1%)	38 (55.9%)	68 (17.8%)	
Education	Middle school and below	10 (38.5%)	16 (61.5%)	26 (6.8%)	0.049^*^
	High school	60 (43.8%)	77 (56.2%)	137 (35.8%)	
	Bachelor's	115 (57.5%)	85 (42.5%)	200 (52.2%)	
	Postgraduate	10 (50.0%)	10 (50.0%)	20 (5.2%)	
Marital status	Single	96 (50.8%)	93 (49.2%)	189 (49.3%)	0.963
	Married	99 (51.0%)	95 (49.0%)	194 (50.7%)	
Family history of obesity	No	45 (36.9%)	77 (63.1%)	122 (31.9%)	<0.001*
	Yes	150 (57.5%)	111 (42.5%)	261 (68.1%)	
Housing	Homeowner	28 (42.4%)	38 (57.6%)	66 (17.2%)	0.108
	Living with f	107 (56.0%)	84 (44.0%)	191 (49.9%)	
	Renter	60 (47.6%)	66 (52.4%)	126 (32.9%)	
Income	0−999	32 (55.2%)	26 (44.8%)	58 (15.1%)	0.397
	1000−4999	23 (42.6%)	31 (57.4%)	54 (14.1%)	
	5000−9999	60 (56.1%)	47 (43.9%)	107 (27.9%)	
	10000−15000	49 (51.6%)	46 (48.4%)	95 (24.8%)	
	Over 15000	31 (44.9%)	38 (55.1%)	69 (18.0%)	
Smoking status	No	147 (52.7%)	132 (47.3%)	279 (72.8%)	0.522
	Previously	14 (46.7%)	16 (53.3%)	30 (7.8%)	
	Yes	34 (45.9%)	40 (54.1%)	74 (19.3%)	
Previous attempts to lose weight	No	73 (44.5%)	91 (55.5%)	164 (42.8%)	0.03*
	Yes	122 (55.7%)	97 (44.3%)	219 (57.2%)	

**Table 3:** Univariate analysis of characteristics associated with WS *Significant values < 0.05 WS: Weight stigma

Variable	p-Value	OR	(95% CI)
Gender			
Male	0.259	0.791	(0.526–1.188)
Female	-	1.00	-
Age group			
18−19	0.936	0.969	(0.452–2.077)
20−29	.038*	0.484	(0.244–0.960)
30−39	.036*	0.460	(0.222–0.952)
40−50	.028*	0.422	(0.195–0.911)
+ 50	.024*	1.00	-
Obesity			
Overweight	.021*	0.288	(0.100–0.829)
Obesity I	<0.001*	0.103	(0.037–0.288)
Obesity II	<0.001*	0.062	(0.023–0.169)
Obesity III	<0.001*	0.026	(0.009–0.076)
Non-obese	<0.001*	1.00	-
Age onset of obesity			
0−10	0.005*	0.494	(0.302–0.808)
11−20	0.383	0.805	(0.494–1.311)
+ 20	0.018*	1.00	-
Employment status			
Unemployed	0.012*	3.897	(1.345–11.293)
Retired	0.082	1.514	(0.949–2.416)
Student	0.049*	1.763	(1.003–3.100)
Current employment	0.022*	1.00	-
Education			
Bachelor	0.520	0.739	(0.294–1.855)
High school	0.603	1.283	(0.502–3.283)
Middle school	0.435	1.600	(0.492–5.207)
Postgrad	0.050	1.00	-
Marital status			
Single	0.963	1.010	(0.676–1.507)
Married	-	1.00	-
Previous attempts to lose weight			
No	0.030*	1.568	(1.043–2.356)
Yes	-	1.00	-
Family history of obesity			
No	0.001*	2.312	(1.486–3.598)
Yes	-	1.00	-
Housing			
Renter	0.058	0.578	(0.329–1.018)
Living with family	0.493	0.811	(0.445–1.478)
Homeowner	0.110	1.00	-
Income (monthly)			
0−999	0.251	0.663	(0.328–1.337)
1000−4999	0.796	1.100	(0.536–2.255)
5000−9999	0.149	0.639	(0.348–1.175)
10000−15000	0.401	0.766	(0.411–1.427)
Over 15000	0.401	1.00	-
Smoking status			
Previous	0.531	1.273	(0.598–2.707)
Yes	0.303	1.310	(0.784–2.191)
No	0.523	1.00	-

Multiple predictors have been included in the logistic regression model to determine whether they are associated with WS. Among the variables considered in the model, both family history of obesity and BMI showed statistical significance. Individuals who did not have a history of obesity in their family showed a higher prevalence of WS than those who did have a family history of obesity (adjusted odds ratio (AOR) = 1.853, 95% CI = 1.126-3.049). Additionally, individuals classified as obesity class III exhibited a lower WS than those without obesity (AOR = 0.027, 95% CI: 0.009-0.08), as indicated in Table 4.

**Table 4:** Multivariate analysis of characteristics associated with WS *Significant values < 0.05 WS: Weight stigma; AOR: adjusted odds ratio; CI: confidence interval

Variable	p-Value	AOR	(95% CI)
Obesity			
Overweight	0.016*	0.272	(0.094–0.786)
Obesity I	<0.001*	0.103	(0.037–0.289)
Obesity II	<0.001*	0.065	(0.024–0.178)
Obesity III	<0.001*	0.027	(0.009–0.080)
Non-obese	<0.001*	1.00	-
Family history of obesity			
No	0.015*	1.853	(1.126–3.049)
Yes	<0.001*	1.00	-

## Discussion

This study examined the effects of sociodemographic factors and BMI on WS. Results demonstrated that WS was significantly associated with sociodemographic factors and BMI in the adult population of Madinah. Regression analysis indicated that the absence of a family history of obesity and BMI were the strongest independent predictors of WS. These findings correspond with a previous similar study showing an association of WS with BMI and family history of obesity [15].

WS and obesity

The multiple logistic regression model showed an inverse relationship between obesity Grade III and WS. Individuals in Class III were the ones least likely to experience WS, and BMI was a negative predictor of WS. These outcomes imply that factors beyond an individual’s control may lead to less stigma toward those with obesity compared with factors that are controllable and cause obesity. People who realize that obesity is caused by uncontrollable factors, such as genetics, are less discriminating and more accepting of individuals with obesity; this finding suggests that the way in which obesity is viewed, and its associated stigma can differ based on the perceived major probable cause of the excess fat [16]. Another explanation of this finding is individuals who accepted their weight were perceived as less stigmatized, had higher self-esteem, and experienced fewer psychological problems than those who did not accept their weight. In simple terms, accepting a person's weight, even for individuals who are obese, is associated with less stigma among others and better perceived mental health [17]. 

Sociodemographic factors and WS

The regression analysis revealed that the lack of a family history of obesity significantly predicted WS. As in the previous study, this finding asserts that family is the most important source of enacted stigma [18]. Having obese family members may provide a protective effect against WS [19]. Individuals without a family history of obesity were more likely to experience higher levels of WS. This suggests that coming from a family background where obesity is absent provides less protection against WS [20]. 

In contrast, as in a previous study conducted by Pearl et al., participants raised by mothers with higher BMIs tended to have lower WS; this indicates that growing up in a family environment where larger body sizes are more normalized can shape more positive attitudes towards one's weight and body. The family appears to be a key social context that either perpetuates or mitigates the development of WS [15]. Moreover, perceiving a family history of being overweight is linked to lower levels of WS and slightly more satisfied with their body shape among overweight women. This indicates that incorporating family-based strategies into weight management programs could be beneficial [20]. 

Although the relationship of WS with age, education, sex, and other factors did not contribute significantly, this could indicate the most predictive factors of WS in the population of Madinah as all these factors could vary according to population [21].

Prevalence of WS

The findings of this study indicate a high prevalence of WS in Madinah, with a rate of 195 (50.9%). These results are closely aligned with a previous investigation conducted in Saudi Arabia, where WS was found to have a prevalence rate of 43.7% in Madinah [11]. Our study specifically targeted the population of Madinah to obtain more precise results. This focused approach enabled a comprehensive analysis of the factors influencing WS within this population, thus offering valuable insights for designing targeted interventions. By concentrating on Madinah, a better understanding of how sociodemographic characteristics and BMI uniquely contribute to WS in this community was gained.

Strength and limitations

As far as we know, such a study has previously not been conducted in Madinah. Some of the studies performed in Saudi Arabia have estimated the prevalence of WS in nationwide samples of adults in Saudi Arabia [8,11]. We were able to gain a more comprehensive understanding of the unique contributions of sociodemographic characteristics and BMI to WS by focusing on the Madinah population. Furthermore, unlike previous similar studies conducted in Saudi Arabia that relied on online self-reported data for weight and height, primary healthcare records were utilized in this study, which enhances the validity and reliability of our findings. Moreover, the study sample was collected from various locations using a careful sampling method to minimize bias and ensure equal representation of different regions.

However, it is essential to recognize that there are several limitations in our study that could potentially influence the results. An important limitation is the use of the median instead of predetermined cutoffs to classify WS. This approach may limit the comparability of our findings with future studies that utilize different criteria to define WS. Another limitation is that the participants were recruited from PHCs, which may not capture individuals with the highest levels of WS. Furthermore, the anthropometric data, including weight and height, were obtained from PHC's electronic records, and the accuracy of these measurements assumes that the data entry processes and equipment were consistently precise and calibrated. However, there is a potential inaccuracy of the anthropometric data due to possible inconsistencies in data entry processes and equipment calibration.

The observed outcome may be influenced by the potential risk of recall bias as the data were obtained via self-reported questionnaires. Individuals seeking treatment from specialized clinics, such as those visiting dietitians or who have undergone bariatric surgery, may experience greater WS. Moreover, the measurement tools used in this study were focused solely on the psychological aspects of WS, rather than incorporating social factors.

Evaluating the social aspects of WS would have yielded more complex insights into the role of social factors in shaping this phenomenon. Consequently, the study is unable to fully capture the complexity of social dynamics that contribute to the stigma associated with weight. Moreover, the measurement tools used did not capture the source of WS, and the study was focused on assessing self-reported experiences of WS.

Future research should examine the potential impact of other family factors, such as childhood experiences and cultural influences, on WS. Furthermore, our model should be applied to a larger sample size using a structural equation modeling approach. Obtaining a comprehensive understanding of these factors can aid in designing more precise interventions to effectively address WS. Having family members with obesity could possibly lead to increased empathy and understanding, thus reducing the likelihood of experiencing WS. Further investigations are required to completely understand the association between family history of obesity and WS. To account for the potential influence of recall bias, future studies can consider employing interview-based methodologies to gather data on WS and its relationship with sociodemographic characteristics. Moreover, a direct measurement by the researcher would have provided more accurate anthropometric data on the study population.

## Conclusions

This study provides evidence that WS is strongly associated with a lack of family history of obesity and is negatively associated with obesity. An individual's obesity status does not solely determine this complex issue. This illustrates the influence of social factors on WS. Comprehending the role of family dynamics and societal norms in shaping an individual's weight status is crucial in managing WS. In addition, addressing WS requires a holistic approach that considers both individual behaviors and external influences.
